# Expression and purification optimization of an N-terminal Pfs230 transmission-blocking vaccine candidate

**DOI:** 10.1016/j.pep.2019.04.001

**Published:** 2019-08

**Authors:** Shwu-Maan Lee, Jordan Plieskatt, Seetha Krishnan, Monika Raina, Rakeshkumar Harishchandra, C. Richter King

**Affiliations:** a PATH's Malaria Vaccine Initiative (MVI), 455 Massachusetts Avenue NW, Suite 1000, Washington, DC, 20001-2621, USA; b Syngene International Ltd, Plot No.2,3,4 &5 Phase IV, Bommasandra Jigani Link Road, Bommasandra Industrial Area, Bangalore, 560099, India

**Keywords:** Baculovirus, Sf9, Malaria, Vaccine, Pfs230, *P. falciparum*

## Abstract

In an effort to control and eventually eliminate malaria, the development of transmission-blocking vaccines has long been sought. However, few antigens have been evaluated in clinical trials, often due to limitations in the expression and purification of the antigen in sufficient yield and quality. Pfs230, a surface antigen of gametocytes, has recently advanced to clinical evaluation as a conjugate vaccine using the *Pseudomonas aeruginosa* exoprotein A carrier protein. Here we continue to build upon prior work of developing a Pfs230 candidate in the baculovirus system, Pfs230C1 (aa 443–731), through systematic process development efforts to improve yield and purity.

Various insect cells including High Five, Sf9 and Super Sf9 were first evaluated for quality and quantity of antigen, along with three insect cell media. In the selection of Sf9 cells, an intact Pfs230C1 was expressed and harvested at 48 h for downstream development. A downstream process, utilizing immobilized metal affinity column (IMAC), followed by ion exchange (IEX) membranes (Mustang S) and finally IEX chromatography (DEAE) yielded a pure Pfs230C1 protein. The complete process was repeated three times at the 20 L scale.

To support the eventual chemistry manufacturing and controls (CMC) of Pfs230C1, analytical tools, including monoclonal antibodies, were developed to characterize the identity, integrity, and purity of Pfs230C1. These analytical tools, taken in combination with the optimized process, were implemented with Current Good Manufacturing Practices (cGMP) in mind with the ultimate objective of Phase I clinical trials.

## Highlights

• Super Sf9, Sf9 and High Five baculovirus cells were evaluated to express the Pfs230 construct.
• Following selection of Sf9 cells to minimize degradation, expression media was optimized.
• A purification approach was developed to produce a pure recombinant product free of host cell proteins.
• A variety of biochemical release assays were developed to support the release and stability of Pfs230.
• A scalable process suitable for cGMP manufacture was developed.

## Graphical abstract

**Figure:**
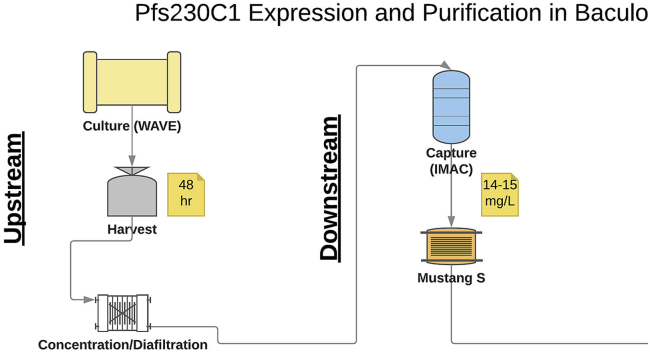

## 1 Introduction

Malaria transmission-blocking vaccines (TBVs) are being evaluated for their potential to serve as a supplemental tool to accelerate the elimination of malaria. They function via the induction of antibodies that block the penetration of the mosquito midgut by sexual stage parasite, thereby breaking the cycle of parasite transmission between human and mosquito hosts [[1], [2], [3], [4]]. Several sexual stage antigens (Pfs25, Pfs230 and Pfs48/45) have been identified as promising TBV targets for research or early stage clinical development. The highly studied TBV antigen, Pfs25, has consistently been associated with poor induction of transmission-blocking antibodies in clinical trials [5]. Nanoparticle delivery has been used in attempts to increase the induction of functional antibodies [6]; however, functional antibody responses were of low magnitude and short-lived [7,8]. These data have contributed to expanded interest in alternative TBV targets, including Pfs230 protein, which is expressed on the surface of gametocytes/gametes [9,10] and serves an essential role in gametocyte biology [11].

The expression and purification of the Pfs230 protein have been challenged by the large size and high degree of complexity of the protein, which is rich in disulfide bonds and contains multiple domains [12]. However, N-terminal fragments of Pfs230 have been successfully expressed in *Pichia* [13], wheat germ [14], plant [15] and Baculovirus [16]. The most clinically advanced Pfs230 candidate (Pfs230 D1M) is produced in *Pichia* [13] and chemically conjugated to *Pseudomonas aeruginosa* exoprotein A (EPA), an immunogenic carrier protein [17]. Clinical trials are underway to test Pfs230-EPA in combination with GSK's proprietary AS01 adjuvant [18] and initial results have been promising [ClinicalTrail.gov Identifier: NCT02942277]. Here, we offer the process development of an alternative Pfs230 candidate, Pfs230C1 in the baculovirus system.

We have previously reported [16] the successful production of the N-terminal fragment of Pfs230 in the baculovirus expression system using Super Sf9 cells. The purified protein (Pfs230C1) was determined to be monomeric with both disulfide bonds properly paired. Further, immunization of mice resulted in the induction of antibodies exhibiting transmission-reducing activity [16]. The biophysical and immunological characterization of Pfs230C1 supported its potential for consideration as a lead candidate to support TBV development.

The baculovirus system has demonstrated scale-up potential and also been used to manufacture commercial vaccines [19]. While the previous study reported on research and discovery aspects of Pfs230C1 [16], characterization from manufacturing and quality control viewpoints was not addressed. In this study, we report the development of a reproducible expression and purification process, as well as effective analytical methods to support the product release and stability, suitable for technology-transfer to a contract manufacturer for cGMP.

## 2 Materials and methods

*Expression and purification of Pfs230C1***.** The Baculovirus expression construct, bacmid and virus for Pfs230C1 was previously described [16] and the final optimized process is described here. Sf9 cells were seeded at 1 × 10^6^ cells/mL in 20 L of SFM4-Insect medium (Hyclone). Multiplicity of Infection (MOI) of one was used to infect 2 million cells/mL in the 20 L Sf9 wave culture. Forty-eight h post infection, the culture was harvested by centrifugation (9000×*g* for 15 min) followed by clarification through 0.8 μm and 0.2 μm filters (Sartopore 2XLG G8, Sartorius). The clarified supernatant was concentrated 10-fold using a 10 kDa tangential flow filtration (TFF) membrane, 0.5 m^2^, (polyether-sulfone, Pall) and diafiltered with 20 mM sodium phosphate, 150 mM NaCl, 100 mM Arginine, pH 7.4 (Buffer A). The concentrate was combined with the membrane wash to a final volume of 4 L and further clarified with 0.8 μm and 0.2 μm filtration (Sartopore 2XLG G7, Sartorius) prior to purification.

The concentrate was loaded onto a pre-equilibrated Ni-Sepharose HP column (GE Healthcare 1.6 × 16cm, 32 mL) at 150 cm/h. The column was washed with 20 column volume (CV) of buffer A followed by 20 CV of 20 mM sodium phosphate, 150 mM NaCl, 150 mM Arginine, pH 7.4. Arginine was removed from the column by washing with 20 CV of 20 mM sodium phosphate, 50 mM NaCl, pH 7.4 (PBS). The target protein was eluted with PBS containing 20 mM imidazole (20 CV) and 45 mM imidazole (20 CV). Subsequent washes with higher concentration (60 and 300 mM) of imidazole removed impurities. The collected eluents were analyzed with SDS PAGE and final pool was selected based on purity.

The immobilized metal affinity column (IMAC) pool (535 mL) was passed through a 10 mL single-use charged membrane (Mustang S, Pall), equilibrated with PBS, at 4 mL/min for further purification. The target protein was chased through the membrane with PBS and collected; impurities were eluted with PBS containing 1 M NaCl.

The flow-through from Mustang S, containing Pfs230C1, was subsequently loaded onto a Fractogel EMD DEAE column (Merck Millipore, 1.5 × 10cm, 17 mL) at 70 cm/h for further purification. The column was washed sequentially with 20 CV each of PBS and PBS containing 140 mM NaCl. The target protein was eluted with PBS containing 270 mM NaCl and the impurities were removed from the column with PBS containing 1 M NaCl. The fractions were analyzed by SDS PAGE to confirm the purity.

The DEAE column elution pool was concentrated twofold, using an Amicon ultra 3 KDa concentrator (Millipore). The concentrate was buffer exchanged into 20 mM HEPES, 150 mM NaCl, pH 7.4 using G-25 (GE Healthcare) column (5 × 20 cm, 400 mL) at flow rate of 30 cm/h. The final pool was concentrated to >1 mg/mL using Amicon 10 kDa, 15 mL concentrator. The concentrated protein was 0.22 μm filtered using a vacuum filter (Stericup, Millipore).

*SDS-PAGE.* For in-process analysis, samples were diluted with 4× LDS (Lithium dodecyl sulfate, Invitrogen) sample buffer, heated at 95 °C for 10 min and loaded in a final volume of 20 μL/well on SDS-PAGE gels (4–12% NuPAGE Bis-Tris, Invitrogen). Gels were run at 150–200 V for 35–50 min in 1× MES SDS running buffer and stained with SimplyBlue™ Safe Stain (Invitrogen).

For final product analysis, DTT was used in reducing samples. Bis-Tris 10% PAGE gels (Novex) were run at 100–150 V for 60 min and Silver stained.

*Production of mouse monoclonal antibody against Pfs230C1.* Five mice were immunized with purified Pfs230C1 in 20 mM Sodium Phosphate, 150 mM NaCl, 5% Glycerol pH 7.0 (Precision Antibody, proprietary immunization protocol). Based on ELISA titer to Pfs230C1, two mice were selected for fusion to produce hybridoma. Pfs230C0 fragment containing Pfs230 amino acid 443–584, expressed in the baculovirus system (unpublished data) was used as a counter antigen. The initial hybridoma screen used ELISA to select Pfs230C1 positive and Pfs230C0 negative clones. Subsequent screening was based on reduction sensitivity on Western blot to select conformational monoclonal antibodies. At least five monoclonal antibodies were produced and all of them recognized the non-reduced form of native Pfs230 from parasite extract in a reductive sensitive manner, implied they recognized native epitopes. One of the conformational monoclonal antibodies, 15A4-1B12, was further used here to characterize Pfs230C1.

*Western blot (Purified protein with anti-his antibody).* Following SDS-PAGE, proteins were transferred onto 0.2 μm nitrocellulose membrane (Pall) and blocked in 5% skim milk in PBS containing 0.05% Tween 20 (PBST) at 5 °C overnight. Primary antibody at a 1:2000 dilution of Penta His antibody (Qiagen) in 1% skim milk in PBST was added and incubated for one hour at room temperature. Membranes were washed with PBST (3× for 5 min each) and secondary antibody, 1:2000 dilution of goat anti mouse IgG-HRP (Santa Cruz) in 1% skim milk (PBST), was added and incubated at room temperature for one hour. Membranes were then again washed with PBST (3× for 5 min each) and developed using TMB (3,3,5,5′-Tetramethylbenzidine, Sigma Aldrich) and hydrogen peroxide.

*Western blotting with parasite extract and Pfs230 using mouse monoclonal antibody.* Mouse monoclonal antibody 15A4-1B12 was tested against parasite extract (1 × 10^6^ parasites per lane) and Pfs230C1 (20 ng) via western blot. The mixture of *P. falciparum* NF54 gametocyte, zygote and ookinete-stage parasites were prepared as described previously [20]. Mouse monoclonal antibody 15A4-1B12 was used at a concentration of 10 μg/mL with a secondary antibody, goat anti-mouse (KPL, Cat# 5220–0310) used at 1:2000.

*Protein concentration.* The Pfs230C1 protein does not contain any tryptophan and therefore A_280_ is not a suitable method for accurate protein determination. The BCA assay was performed on microtiter plates for both in process and final product analysis to determine the protein concentration of Pfs230C1 [21].

*SE (Size Exclusion)-HPLC.* SE-HPLC analysis of purified Pfs230C1 was performed on a BioAssist G3SWxl column (7.8 × 300 mm, TOSOH Biosciences, King of Prussia, PA) operated at column temperature of 30 °C on an Agilent 1260 HPLC system. The mobile phase consisted of 200 mM sodium phosphate pH 6.8 with a flow rate of 0.7 mL/min with detection at absorbance of 214 nm. Gel filtration standards (Bio-Rad, Hercules, CA) were used to confirm system suitability as well as confirmation of expected retention time of Pfs230C1 (e.g. between the 158kda and 44 kDa marker). Pfs230C1 molar mass via SE-HPLC was previously confirmed as 35.2 kDa [16].

*Ion-Exchange (IEX)-HPLC.* IEX-HPLC analysis of purified Pfs230C1 was performed on a TSK-Gel BioAssist Q column (4.6 × 50mm, 10 μm, TOSOH Biosciences) at 25 °C on an Agilent 1260 HPLC system. The mobile phase consisted of a gradient of A) 10 mM sodium phosphate pH 7.4 and B) 10 mM sodium phosphate pH 7.4, 1 M NaCl at a flow rate of 0.7 mL/min (Table 1).

**Table 1:** Ion-Exchange HPLC Gradient for purity determination of Pfs230C1.

Time (min)	Buffer A (%)	Buffer B (%)
0	90	10
5	90	10
7	70	30
20	50	50
22	40	60
30	30	70
35	0	100
37	0	100
39	90	10

*Reverse phase (RP)-HPLC.* RP-HPLC was performed on a 5 μm Bio basic phenyl column, 2.1 × 150 mm (Thermo scientific) at 25 °C at a flow rate of 0.2 mL/min. Mobile phase A contains milliQ water with 0.1% TFA and mobile phase B contains 100% acetonitrile in 0.1% TFA. The gradient is shown in Table 2.

**Table 2:** Reverse phase HPLC gradient for purity determination of Pfs230C1.

Time (min)	Buffer A (%)	Buffer B (%)
0	90	10
5	90	10
6	70	30
25	50	50
26	1	99
29	1	99
31	90	10
36	90	10

*LC/Intact mass.* Pfs230C1 was separated on a reversed phase Accucore-150-C4, 100 × 2.1 mm, 2.6 μm column at a column temperature of 60 °C, using an UHPLC (Waters Corporation) with solvents A (99.9% H_2_O, 0.1% formic acid) and B (99.9% acetonitrile, 0.1% formic acid) over a gradient from 10 to 99% B over 29 min at a flow rate of 0.2 mL/min. Loading volume was 30 μL of prepared protein and wavelength monitored at 214 nm. The intact mass of the protein was measured using a Waters SYNAPT G2 (Waters Corporation, Milford, MA). A second generation high definition Mass Spectrometry™ (HDMS™) of exact mass MS/MS platform which provides Collision Induced Dissociation (CID) fragmentation with high resolution and accurate mass measurements with 5 ppm mass accuracy.

*N-terminal sequencing.* Theoretical masses for N-terminal amino acids of Pfs230C1 (DEYVDEK, m/z 897.9 Da) were computed for predicting the b & y ions generated during MS/MS analysis. Pfs230C1 was digested with sequencing grade Lys-C (Wako Cat no: 125–02543) and sequentially subjected to CID. In order to gain N-terminal structural information from the fragment ions generated, resulting MS2 fragment ions having the amino acid sequence and molecular mass (DEYVDEK) of 897.9 Da were captured. Corresponding MS/MS data for this peptide having amino acid sequence “DEYVDEK” was confirmed with 5-ppm tolerance for fragment ions of both b and y ions. This resulting fragmented ions provides complete N-terminal sequence coverage.

*Host cell protein (HCP).* HCP was measured via an ELISA developed from the Sf9 insect cell HCP assay kit (Cygnus, catalog #F840). Initially, different dilution factors were evaluated to determine the linear range. Once the dilution range was defined, spike recovery was performed. At 20–40-fold dilution, the recovery was 100 ± 10% resulting in a working range of 3–100 ng/mL HCP.

*Host cell DNA.* To measure residual DNA, prepSEQ residual DNA sample preparation kit (Applied Biosystems) was used to extract host cell DNA from samples containing Pfs230C1 via chemical lysis and magnetic beads. The extracted DNA was quantified using picogreen dye (Quanti-it picogreen ds DNA assay kit from Life technologies) in microtiter plates. The standards ranged from 200 to 2500 pg/well, and spike-recovery was determined to be 100 ± 20%.

*Residual nickel***.** Inductively Coupled Plasma Mass Spectrometry (ICP-MS) with certified nickel standard (Inorganic venture, CGNI1-1000 mg/L, Christiansburg, Virginia) was used to quantify residual nickel in the purified Pfs230C1. At linear range of 0.5–10 ppb, the recovery of standard was within 100 ± 15%. The amount of nickel in the sample was found to be below the standard range at 1:1000 dilution. Residual nickel was reported as <0.5 ppm.

*Kinetic endotoxin assay.* Spectramax plus spectrophotometer (kinetic endotoxin assay) was used to quantify endotoxin content of purified Pfs230C1 with EndoSafe Endotoxin (Charles River), EndoSafe Lysate (Charles River), and EndoSafe LAL Reagent Water (Charles River) according to manufacturer's procedures.

*Bioburden assay***.** To measure bioburden, USP<61> was followed with a membrane filtration system (Millipore) using 1 mL test samples.

## 3 Results

**Fig. 1:**
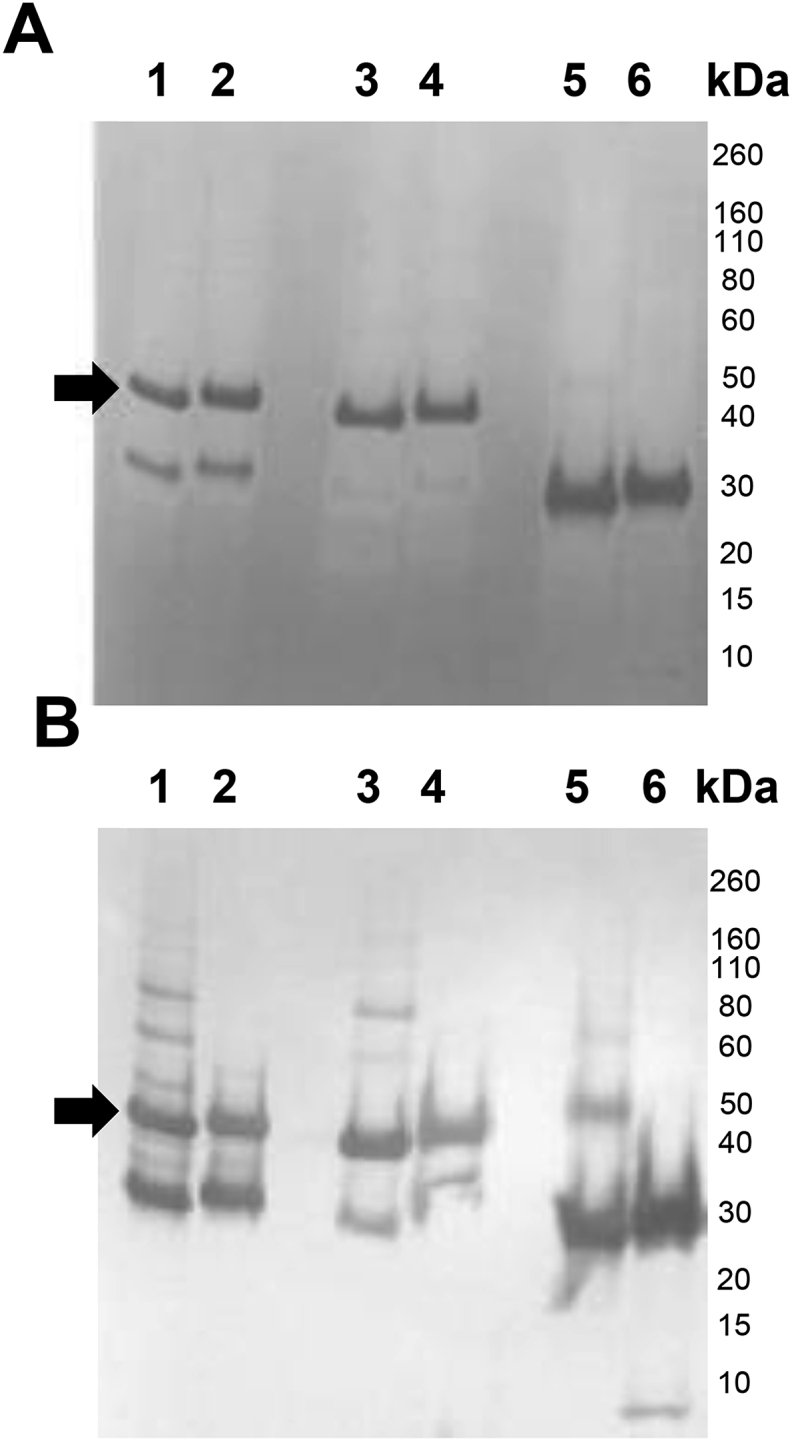
**(A)** SDS-PAGE and **(B)** Western blot (anti-his antibody) analysis of purified Pfs230C1 from Super Sf9 (Lanes 1–2), Sf9 (Lanes 3–4) and High Five cells (Lanes 5–6). Samples loaded under non-reducing (Lanes 1, 3, 5) and reducing (Lanes 2, 4, 6) conditions. Arrows indicate the target Pfs230C1 protein.

*Optimization of Pfs230C1 upstream process: Cell line comparison.* The performance of three cell lines, Super Sf9, Sf9 and High Five, was first compared for the expression level and quality of expressed and secreted recombinant protein Pfs230C1. This comparison study was conducted in 1 L cultures using ESF 921 medium (Expression Systems, CA, USA) in 5 L wave bags with one million insect cell/mL infected at one MOI. The cell density and percent viability were comparable for all the three cell lines (Table 3), with a noted viability drop upon infection. The culture was harvested at 72 h, concentrated, diafiltered and evaluated on a His60 Ni Superflow (Clontech) column (0.68 × 10 cm, 3 mL) to determine protein quality and yield of Pfs230C1 from the supernatant using SDS-PAGE and Western blot (Fig. 1). The yield for Super Sf9, Sf9 and High Five was 23, 7 and 63 mg/L culture respectively. However, the expressed protein from High Five cells was mostly degraded, and, from Super Sf9 it showed partial degradation. Based on Western blot analysis (Fig. 1B), it was determined that the degradation resulted in an N-terminal truncated protein, as the remaining fragment was recognized by anti-his (his tag present on the C-terminus). The selection of Sf9 cells as the preferred substrate was based on the quality and integrity of the expressed Pfs230C1 product and the regulatory history of Sf9 cells [22].

**Table 3:** Cell density and viability of Sf9, Super Sf9, and High Five cells expressing Pfs230C1.

Time (h)	MOI	Cell density x10^6^ cells			Viability		
		Sf9	Super Sf9	Hi5	Sf9	Super Sf9	Hi5
0	1	1.0	1.0	1.0	98%	98%	98%
24	1	1.4	1.25	1.25	95%	96%	96%
48	1	1.1	1.05	1.05	84%	87%	87%
72	1	0.78	0.68	0.68	59%	60%	60%

**Fig. 2:**
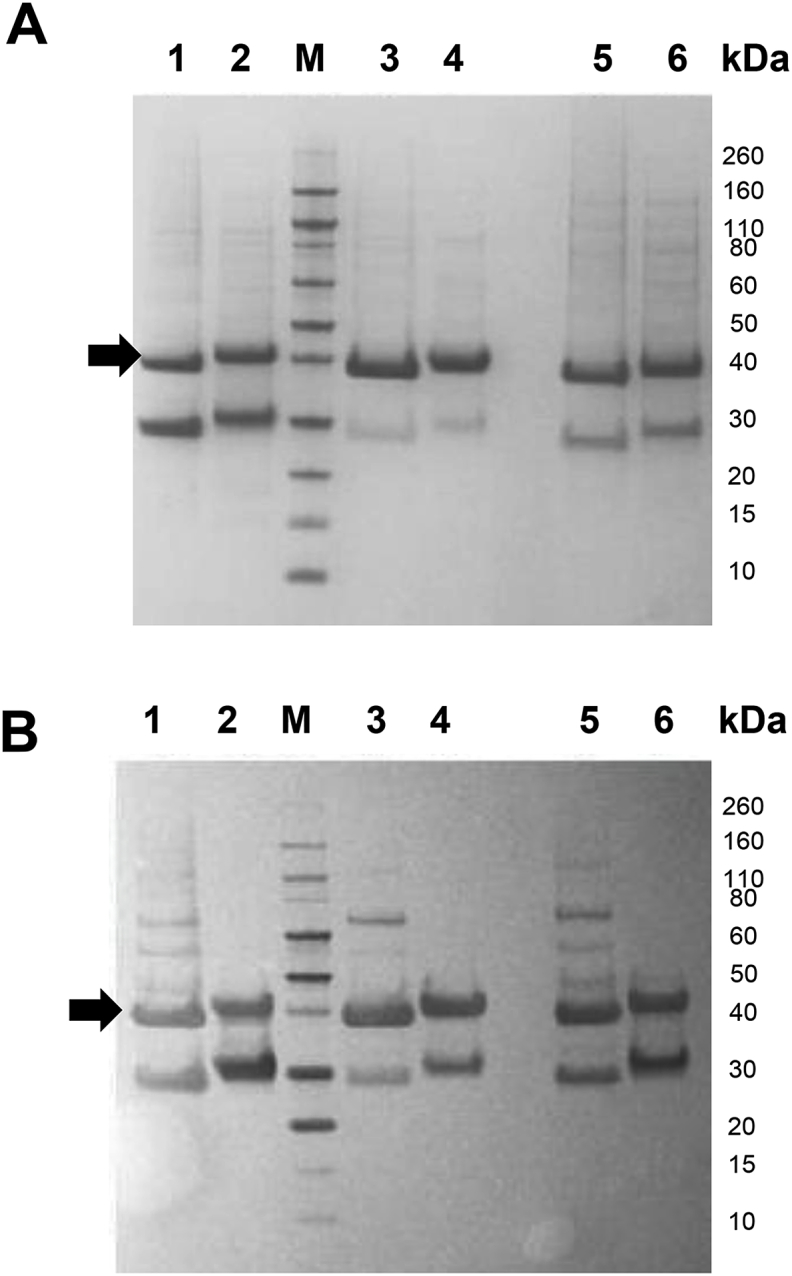
**(A)** SDS-PAGE and **(B)** Western blot (anti-his) analysis of the purified Pfs230C1 protein for the three culture media at 72 h harvest. SFM4 (Lanes 1–2), ESF921 (Lanes 3–4), and Ex-cell 420 (lanes 5–6) were evaluated under non-reducing (Lanes 1, 3, 5) and reducing (lanes 2, 4, 6) conditions. (M) denotes marker lane. Arrows indicate the target Pfs230C1 protein.

*Optimization of Pfs230C1 upstream process: Expression Media Comparison.* Using a similar approach, three media were evaluated with the selected Sf9 cells to further optimize the expression and quality of the target protein. Cell count and cell viability (Table 4) decreased significantly between 48 and 72 h and the resulting supernatant was analyzed by SDS-PAGE and Western (Fig. 2). At 72 h harvest, the purified protein yield was 18, 7 and 7 mg/L culture for SFM4 (Hyclone), ESF921 (Expression Systems) and EX-CELL 420 (Sigma-Aldrich) media, respectively. However, all three media were associated with degradation following SDS-PAGE and Western blot analysis (Fig. 2), most likely as a result of decreased cell viability (Table 4). Yields were calculated as previously described using a single IMAC purification; this yield calculation included the degradation products that bound to IMAC column.

**Table 4:** Cell density and viability of Sf9 cells evaluated in three media for the expression of Pfs230C1.

Time (h)	MOI	Cell density x10^6^ cells			Viability		
		SFM4	ESF921	Ex-Cell 420	SFM4	ESF921	Ex-Cell 420
0	1	1.0	1.0	1.0	96%	95%	98%
24	1	1.2	1.0	0.90	91%	98%	98%
48	1	0.91	0.98	0.79	80%	92%	81%
72	1	0.30	0.95	0.22	28%	82%	30%

To improve protein quality and re-evaluate yield, the culture time was shortened to 48 h. At 48 h culture time, the protein degradation was significantly reduced and SFM4 provided the highest calculated yield (6 mg/L) among the three media evaluated (data not shown). SFM4 was selected for the upstream process with further optimization of cell density and MOI.

*Optimization of Pfs230C1 upstream process: Cell Density and MOI.* In the next stage of the upstream optimization, four different cell densities and two different MOIs were evaluated using the selected Sf9 cells and SFM4 medium. The cell density, viability and yield from all of the five conditions are presented in Table 5. There was no observable increase yield with increased cell density beyond two million cells or MOI above one. Pfs230C1 yields were calculated as previously described using single step IMAC purification. The following conditions were selected to support upstream process development: 2 million/mL Sf9 cells seeded in SFM4 with MOI of one and harvest at 48 h.

**Table 5:** Expression yield with four cell densities and two MOIs at 48 h harvest.

Cell density at infection	MOI	Cell density at 48 h	Viability at harvest	Yield
1 × 10^6^ cells	1	0.9 × 10^6^ cells	86%	8.3 mg/L
2 × 10^6^ cells	1	1.8 × 10^6^ cells	83%	21.2 mg/L
4 × 10^6^ cells	1	3.6 × 10^6^ cells	79%	11.2 mg/L
8 × 10^6^ cells	1	6.9 × 10^6^ cells	72%	10.5 mg/L
8 × 10^6^ cells	3	6 × 10^6^ cells	68%	9 mg/L

**Fig. 3:**
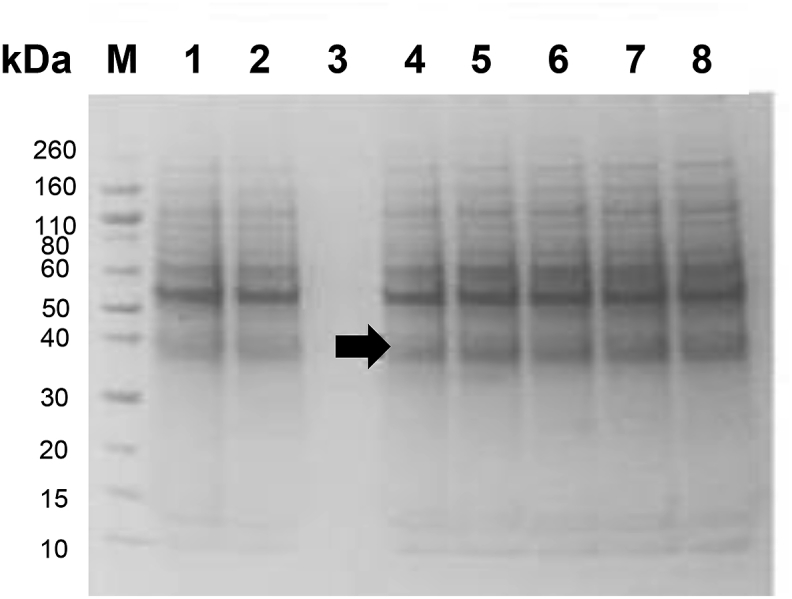
SDS-PAGE analysis of Pfs230C1 five-fold concentrate freeze and thaw analysis. Concentrated supernatant before (Lane 1) and after (Lane 2) diafiltration. Diafiltrate permeate (Lane 3). Concentrated and diafiltered Pfs230C1 at 1 h 4 °C (Lane 4) and −80 °C (Lane 5). Subsequent analysis of supernatant after one, two and three freeze thaw/cycles (Lanes 6–8 respectively). All samples run under non-reducing conditions. Arrow indicate the target Pfs230C1 protein.

*Optimization of Pfs230C1 upstream process at 20 L.* A 20 L production batch was manufactured in a wave bioreactor using the optimized fermentation parameters and scaled appropriately from 2 L. The harvest was concentrated five-fold, buffer exchanged and divided into aliquots to provide consistent starting material for downstream development. A three-cycle freeze thaw study was also performed to assess a potential process hold-step. The resulting Pfs230C1, as evaluated by SDS-PAGE (Fig. 3), show no changes (aggregation of degradation) through freeze/thaw (Fig. 3). This holding point study decoupled upstream and downstream processes and provided flexibility in scheduling for the future manufacturing, should the two processes not be able to occur consecutively.

*20 L Downstream Process.* The optimization of downstream development involved resin screen and abbreviated study of pH, salt, and column conditions to produce a pure, stable Pfs230C1 recombinant protein (data not shown). Further, scalability and cGMP compatibility were considered when screening column steps (e.g. column diameter, bed-height) and process steps (e.g. volume of eluates, etc.). The optimized downstream process is comprised of six stages, involving three column steps (Table 6). This process, in brief, includes: a metal affinity column, an ion-exchange membrane and an ion-exchange column (Table 6).

**Table 6:** Six stages of the optimized Pfs230C1 downstream process. Product is reported as total protein concentration per L of fermentation including product-derived impurities.

Stage	Steps	Description	Product
1	UF/DF	The culture harvest is concentrated 10-fold using a 10 kDa membrane, buffer exchanged into IMAC column loading buffer, and 0.22 μm filtered. This step reduces process volume by fivefold.	ND

2	IMAC purification	The concentrate is loaded onto a Ni-Sepharose HP column (1.6 × 16 cm, 32 mL), the his-tagged Pfs230C1 binds to the column while most of the impurities flow through, high molecular weight impurities are subsequently washed away with 100 mM and 150 mM arginine, The target protein is eluted with a stepwise increase of imidazole at pH 7.4.	14–15 mg/L

3	Mustang S	The elution pool from IMAC column is passed through a 10 mL Mustang S membrane. The negatively charged Pfs230C1 flows through at pH 7.4, while the aggregates and impurities bind to the membrane.	14 mg/L

4	Fractogel EMD DEAE	The Mustang S flow-through (containing Pfs230C1) is loaded onto a Fractogel EMD DEAE column (1.5 × 10 cm, 17 mL), washed and target protein eluted with a stepwise increase of NaCl at pH 7.4.	11–13 mg/L

5	UF	An optional concentration is performed on the DEAE eluate to reduce the process volume.	

6	G-25	G-25 column (5 × 20cm, 400 mL) is used to exchange the protein into the formulation buffer (20 mM HEPES, 150 mM NaCl, pH7.4). The formulated Pfs230C1 is concentrated to 1 mg/mL target, 0.2 μm filtered and stored as frozen aliquots at ≤ - 60 °C.	9–12 mg/L

**Fig. 4:**
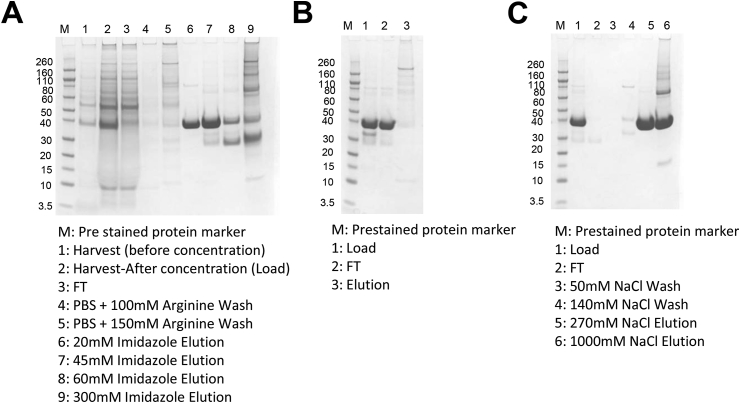
SDS-PAGE analysis of Pfs230C1 during downstream purification. (A) Nickel Sepharose purification column, (B) Mustang S membrane chromatography, and (C) DEAE chromatography. All samples run under non-reducing conditions.

During process optimization, it was determined that including 100 mM and 150 mM arginine with PBS as two sequential washing steps effectively removed high molecular weight impurities [23] without significant loss of the target monomeric Pfs230C1 (Fig. 4A, lanes 4–5). The target protein was eluted with 20–45 mM imidazole. At 60 mM imidazole, the degradative product was eluted and at 300 mM imidazole, high MW impurities were eluted.

The next step of purification used a Mustang S membrane. The target protein has a theoretical pI of 4.77 and it is negatively charged at pH of 7.4. Consequently, it flowed through the membrane and impurities were bound and subsequently removed (Fig. 4B).

The final polishing step used anion exchange, the target protein bound to Fractogel EMD DEAE at pH 7.4 and was eluted with a stepwise increase of salt concentration. High and low molecular weight impurities were eluted after the target protein (Fig. 4C). The DEAE elution is concentrated and subsequently buffer-exchanged utilizing gel-filtration chromatography (G-25). Such steps may be further optimized during technology-transfer and scale-up such as UF/DF to conduct buffer-exchange in a single-step. From three process consistency runs the complete 20 L process (upstream and downstream) yielded 200 ± 50 mg or ∼ 10 mg/L purified Pfs230C1.

**Fig. 5:**
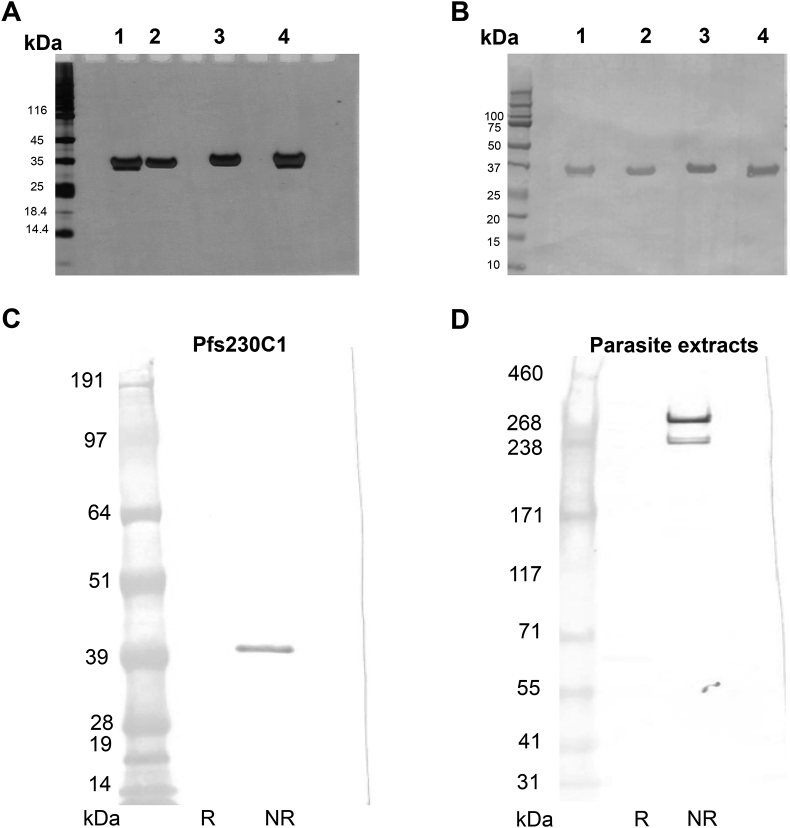
SDS-PAGE and Western blot analysis of final purified Pfs230C1. (A) SDS-PAGE under non-reducing and reducing conditions followed by silver stain. (B) Western blot with anti-his antibody. (A–B) Lanes 1–2 representative Pfs230C1 from the original Super Sf9 process [16], and Lanes 3–4 Pfs230C1 derived from 20 L optimized process. Non-reducing (Lanes 1, 4) and reducing (Lanes 2, 3) conditions. Western blot analysis of (C) Pfs230C1 and (D) native parasite extracts with mouse monoclonal antibody 15A4-1B12 under reducing (R) and non-reducing (NR) conditions. Different molecular weight marker standards are utilized for panels C and D given the difference in molecular weight of target protein. Expected molecular weight of native Pfs230 is ∼363 kDa, however, since protease inhibitors are not utilized, multiple bands are visible.

*Pfs230C1 is a well characterized and consistent product from baculovirus.* The batch analysis of the three consistency lots are summarized in Table 7. The purified protein was a clear, colorless solution at neutral pH. Further, SDS-PAGE shows a dominant band close to the expected molecular weight around 33 kDa (Fig. 5A) and Western blot using anti-his confirmed that the C-terminal histidine tag was intact (Fig. 5B).

**Table 7:** Batch analysis of the three Pfs230C1 consistency lots.

Quality Attributes			Pfs230C1 Lot 1	Pfs230C1 Lot 2	Pfs230C1 Lot 3
Visual appearance			Clear colorless solution	Clear colorless solution	Clear colorless solution
pH			7.52	6.99	7.24
SDS PAGE			Single band	Single band	Single band
Western (anti-his)			Single band	Single band	Single band
Total Protein (mg/mL)			0.92	1.12	1.12
SEC		HMW (%)	0.2	0.2	0.0
		Monomer (%)	98.2	94.9	96.7
		LMW (%)	1.6	4.9	3.3
IEX - HPLC		Pre-Peak (%)	0.2	1.1	0.6
		Peak 1 (%)	53.3	63.1	58.9
		Peal 2 (%)	33.3	31.4	35.2
		Post-peak (%)	13.3	4.5	5.4
RP-HPLC		Pre-Peak (%)	1.8	4.4	1.6
		Peak 2 (%)	68.7	63.2	63.8
		Peak 1(%)	25.9	28.4	29.8
		Post peak (%)	3.5	4.0	4.7
Intact mass		Non-reduced	33510 Da	33510 Da	33510 Da
		Reduced	33514 Da	33514 Da	33515 Da
N-terminal sequence			DEYVDEK	DEYVDEK	DEYVDEK
HCP (ppm)			155	341	175
Host cell DNA (ppm)			6.0	3.3	5.8
Residual Ni (ppm)			<0.5	<0.5	<0.5
Endotoxin (EU/mL)			<0.25	<0.25	<0.25
Bioburden	TAMC (CFU/mL)		<1	<1	<1
	TYMC (CFU/mL)		<1	<1	<1

TAMC: total aerobic microbial count; TYMC: total yeast and mold count.

A panel of mouse monoclonal antibodies were raised against purified Pfs230C1, and these were screened for reactivity to reduction sensitive epitopes using Pfs230C1 and native parasite extract (Data not shown). One antibody, mAb 15A4-1B12, was selected for characterization of Pfs230C1. In western blot analysis, the 15A4-1B12 mouse monoclonal antibody reacted with both native parasite extracts, consistent with binding to full length Pfs230, as well as recombinant Pfs230C1 under non-reducing conditions (Fig. 5C and D). It should be noted, since a protease inhibitor is not used in the native extract preparation, degradant bands often result from the expected ∼363 kDa band as seen by others [24]. This data confirmed the presence of a native epitope on the purified Pfs230C1 protein.

**Fig. 6:**
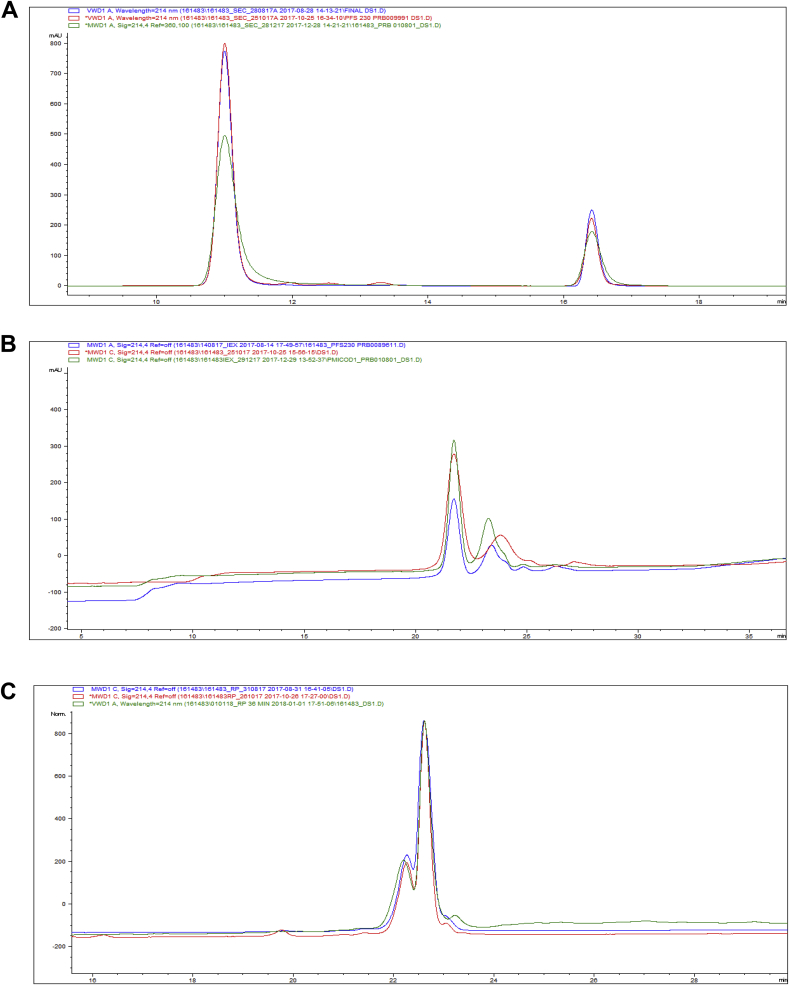
(A) Size exclusion HPLC analysis (B) Ion exchanged HPLC analysis and (C) Reverse Phase HPLC analysis of three 20 L consistency lots preparation of Pfs230C1. Blue trace indicates Lot #1, red trace indicates Lot #2 and green trace indicates Lot #3 of final purified Pfs230C1. In SE-HPLC analysis of Pfs230C1 (Panel A), second peak is absorption peak of buffer components (Data not shown).

To supplement the purity analysis of SDS-PAGE with an orthogonal method, SE-HPLC was employed. A typical SE-HPLC profile is shown in Fig. 6A for Pfs230C1. Previously it was shown via multiangle light scattering [16] that Pfs230C1 migrates as a monomer (approximately 35 kDa) via SE-HPLC and between the molecular weight markers of 158 kDa and 44 kDa with an expected retention time of 11 min. Analysis by SE-HPLC here, showed a purity of at least 95% and absence of significant aggregation or degradation.

In addition, IEX- and RP-HPLC were used to discern purity and potential glycosylated forms of Pfs230C1 present in the final purified protein. IEX-HPLC separated Pfs230C1 into two main peaks, the non-glycosylated form eluted first (peak 1) followed by the glycosylated form (peak 2) (Fig. 6B). The identification of peak 1 and peak 2 was confirmed by LC/MS (data not shown). The ratio of non-glycosylated form to glycosylated form was approximately 2:1, which remained consistent for the three consistency lots (Table 7, Fig. 6B.).

RP-HPLC also discerned Pfs230C1 as two peaks with the glycosylated form more hydrophilic and eluted first, followed by the non-glycosylated form (Fig. 6C). Despite of not achieving base line resolution, LC/MS confirmed the identity of the peak 1 and peak 2 (data not shown). The ratio of non-glycosylated form (peak 2) to glycosylated form (peak 1) was again approximately 2:1 as reported by RP-HPLC and consistent for the three consistency lots (Table 7, Fig. 6C).

The results of intact mass determination were consistent with the theoretical mass of the protein (±1Da) and the reduction of the two disulfide bonds increased the mass by the expected 4 Daltons (Table 7). The mass of the glycosylated form was consistent with that previously reported [16] (data not shown).

The first seven N-terminal amino acids (DEYVDEK) of Pfs230C1 reported in Table 7 were determined by LC/MS/MS of the first LysC digested peptide with a molecular mass of 897.9 Da. This result was consistent with the N-terminal aspartic acid reported earlier [16].

Process residuals and impurities were evaluated with assays to detect HCP, host cell DNA, residual nickel, and endotoxin. The purified Pfs230C1 samples contain <500 ppm of HCP, <10 ppm host cell DNA, <0.5 ppm residual nickel, and low endotoxin (Table 7). This level of residual DNA would allow a maximum patient dose of 1 mg purified Pfs230C1 protein, well above desired doses, based on WHO TRS-87 limit of 10ng/dose. Residual nickel was reported as lower than the limit of detection (<0.5 ppm).

**Fig. 7:**
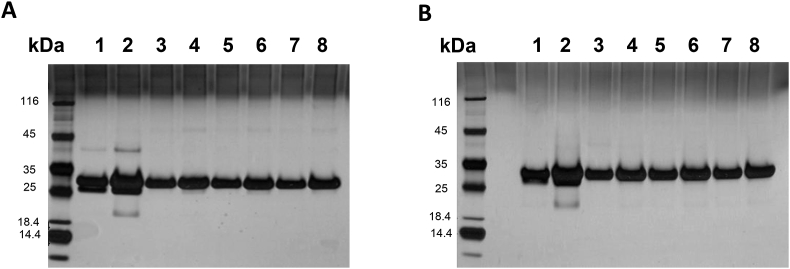
SDS-PAGE Freeze/thaw stability analysis of final purified Pfs230C1 under non-reducing (A) and reducing (B) conditions followed by silver stain. Reference Pfs230C1 from previous [16] Super Sf9 process (Lanes 1–2). Pfs230C1 from 20 L optimized process after one (Lanes 3–4) three (Lanes 5–6) and five (lanes 7–8) freeze/thaw cycles. Proteins loaded at 1 μg (Lanes 1, 3, 5, 7) and 5 μg (Lanes 2, 4, 6, 8).

*Stability of Pfs230C1.* Five freeze/thaw cycles were studied using the third consistency lot as the starting material. There were no changes in SDS-PAGE, Western blot, SEC-, IEX- and RP- HPLC profiles (full data not shown). Pfs230C1, as detected by SDS-PAGE with silver stain (Fig. 7), showed no change over one-, three-, and five-freeze/thaw cycles. Material from the original super Sf9 process [16] serves as a reference standard (Fig. 7 Lanes 1–2), with noted minor bands evident at higher protein loads (Fig. 7 Lane 2), also indicating the process developed here at 20 L results in a protein of better purity and stability than previously reported.

## 4 Discussion

To support the continued development of a malaria transmission-blocking vaccine, we report an optimized, scalable, and reproducible process to produce Pfs230C1 expressed in baculovirus. A final yield of 10 mg/L is reported, suitable to readily produce greater than one gram of purified protein, assuming a five-fold scale-up for the current 20 L process, and sufficient to support GLP toxicology and Phase I clinical trials, the primary objective of the process developed here. It should be noted that further optimization would be required to obtain the yields and scales required to support mass deployment of a malaria TBV and/or commercialization. However with two commercialized baculovirus products [25,26], it is anticipated that the interest and capacity exists [19] if the Pfs230C1 product proves efficacious in clinical trials.

In a systematic evaluation and optimization of the upstream process, three cell lines were evaluated to optimize the expression, integrity and quality of the Pfs230C1 protein from baculovirus. High Five cells are a clonal isolate derived from the parental *Trichoplusia ni* cell line (cabbage looper ovary) and Sf9 cells are derived from the pupal ovarian tissue of the fall armyworm *Spodoptera frugiperda*. Super Sf9 is a genetically engineered Sf9 subclone (Oxford Expression Technologies). These cell lines are commonly used for the expression of recombinant proteins using the Baculovirus Expression Vector System and the Sf9 derived cell clone designated Sf+ is used to produce Flublok by Protein Sciences Corp, now Sanofi [22]. A High Five derived Rix4446 cell line is used by GlaxoSmithKline to produce a cervical cancer vaccine, Cervarix [26]. The High Five cell line was reported to achieve higher titers of recombinant proteins compared with Sf9 on the per cell basis. However, the Sf9 cells commonly achieve higher densities in culture than do High Five cells and therefore provide higher volumetric productivities than the High Five [27]. While we observed higher titer of recombinant Pfs230C1 per cell basis in High Five cells, we also observed higher proteolytic activities in High Five cells. Therefore, Sf9 cells were selected based on product quality for continued development. However, future work may evaluate earlier harvest time points, where cell viability may be higher, and proteolytic activity lower.

Insect cell growth media have evolved significantly in the past five decades, from basal media supplemented with hemolymph or animal serum, to highly optimized serum-free media and feeds capable of supporting very high cell densities and recombinant protein yields. The substitution of animal sera with protein hydrolysates in serum free medium results in greatly reduced medium costs and much improved process scalability [28].

Three serum-free commercial insect media were used in the evaluation of Pfs230C1 in the Sf9 cell line. SFM4 provided the highest yield, at both 48 h and 72 h harvest, and was our medium of choice for further work. Further, SFM4 is free of animal cell components, significantly reducing the chances of introducing an adventitious agent during manufacturing [28]. In the current studies, we demonstrated that SFM4 medium can support high density culture at 8 million cells/mL; however, we did not achieve higher yield of Pfs230C1 at increased MOI.

The fermentation parameters (cell density, medium, MOI, harvest time) may further be optimized using Design of Experiments to study interaction of individual critical parameters in later studies.

The downstream processing employed a series of scalable steps including an initial UF/DF reduced processing volume thereby decreasing loading time onto the IMAC capture column. The IMAC column substantially enriched the target protein, and significantly reduced impurities for subsequent polishing steps. Two additional polishing steps, Mustang S and DEAE, improved purity and removed process residuals.

There was low level of undesirable *O*-glycosylation previously reported for Pfs230C1 [16] and while the glycosylation did not appear to impact induction of functional antibodies [16], it was important to report a consistent glycosylation profile across consistency runs. Here, we developed two orthogonal HPLC methods to monitor the ratio of non-glycosylated form vs glycosylated form and found the ratio to be consistent across the three consistency runs. Further work may explore alternative sequence or construct designs for the Pfs230C1 gene to limit glycosylation. In addition, while the native parasite does not contain glycosylation machinery [29] and therefore the native antigen is expected to be non-glycosylated, the partial presence of glycosylated forms of Pfs230C1 (as prepared here from baculovirus) does not appear to impact the ability of the recombinant Pfs230C1 protein to induce functional antibodies [16]. However, further work would need to be completed to separate the glycosylated from non-glycosylated form and evaluate the ability of each form alone to induce functional antibodies to confirm such statements.

While Pfs230C1 is stable after multiple freeze-thawing cycles, studies are in progress to evaluate the long-term stability of the Pfs230C1 generated through these processes. Currently, no instability has been seen through storage at ≤ −60 °C for up to nine months. However, the Pfs230C1 protein will benefit from a systematic formulation study to maintain stability at 2–8 °C and 25 °C. Evaluation of adjuvant compatibility and potential pharmacy manipulations is also required. The importance of studying adjuvant formulation is indicated by results indicating that for Pfs230C1, aluminum hydroxide (Alhydrogel^®^) formulation induced only marginal functional activities in the murine model [30]. Therefore the compatibility and immunogenicity of Pfs230C1 with alternative adjuvants and carriers is ongoing and includes QS-21 [31], GLA-LSQ [32], and the CoPoP liposome [33,34].

In conclusion, we developed a scalable process to produce Pfs230C1 in a baculovirus expression system and optimized both upstream and downstream manufacturing steps suitable for initial clinical evaluation. It is likely that further development, some of which is underway, including 1) exploration of sequence alteration to avoid glycosylation, 2) development of a non-his-tagged variant, and 3) Further optimization/DOE to improve yields would be required if the Pfs230C1 provided efficacious as a TBV in initial clinical evaluation. The current process as developed here produces a protein of sufficient purity and stability that could be formulated with a suitable adjuvant. We have also developed, and reported here, the analytical methods to determine the quality (identity, purity, and integrity) of the purified Pfs230C1 protein and monitor the stability to support preclinical and initial clinical studies.

## Declarations of interest

None

## Funding

This work was supported in whole or in part by a grant from the Bill & Melinda Gates Foundation, Seattle, WA. The mouse immunization and generation of monoclonal antibodies were supported in part by the Intramural Research Program of NIAID, NIH.

The views expressed herein are solely those of the authors and do not necessarily reflect the views of the Bill & Melinda Gates Foundation.
